# Neuroimaging Characteristics of Pruritus Induced by Eczema: An fMRI Study

**DOI:** 10.1002/brb3.70415

**Published:** 2025-03-23

**Authors:** Tae‐eun Kim, Jin Li, Larissa Tao, Ji‐ming Tao, Xiang‐yu Wei

**Affiliations:** ^1^ Department of Acupuncture Shuguang Hospital Affiliated to Shanghai University of Traditional Chinese Medicine Shanghai China; ^2^ International Education College Shanghai University of Traditional Chinese Medicine Shanghai China; ^3^ Department of Rehabilitation Medicine Shuguang Hospital Affiliated to Shanghai University of Traditional Chinese Medicine Shanghai China

**Keywords:** eczema, fraction amplitude of low‐frequency fluctuation, functional connectivity, functional magnetic resonance imaging, pruritus

## Abstract

**Objective:**

To explore the neuroimaging characteristics of eczema‐induced pruritus with resting‐state functional magnetic resonance imaging (rs‐fMRI).

**Methods:**

A total of 42 patients with eczema were recruited in the PE group, and 42 healthy participants were included in the HC group. The Visual Analogue Score (VAS), 12‐Item Pruritus Severity Scale (12‐PSS), Pittsburgh Sleep Quality Index (PSQI), and Self‐Rating Anxiety Scale (SAS) were recorded in the PE group. The different values of fraction amplitude of low‐frequency fluctuation (fALFF) and functional connectivity (FC) were compared after rs‐fMRI scanning.

**Results:**

Compared with the HC group, the fALFF values of the left precentral gyrus, left postcentral gyrus, left supplementary motor area (SMA), and left midcingulate cortex in the PE group were increased. The FC values between the left precentral gyrus, bilateral superior temporal gyrus, bilateral hippocampus, and left inferior occipital gyrus in the PE group were decreased. The FC values between left SMA and bilateral superior temporal gyrus in the PE group were decreased. The 12‐PSS score was positively correlated with fALFF value of the left precentral gyrus and left postcentral gyrus.

**Conclusion:**

Pruritus caused increased spontaneous activity in given cerebral regions, involving the perception of itch, control of scratching movements, and expression of itch‐related emotions. Meanwhile, there is a correlation between fALFF values of given cerebral regions and clinical scales, which provided potential neurobiological markers for the future study of pruritus.

## Graphical abstract

Patients with eczema‐related pruritus showed increased spontaneous neural activity in brain regions linked to itch perception, scratching behavior regulation, and emotional responses to itch, compared to healthy participants. This heightened neural activity suggests altered brain processing in response to chronic itch.

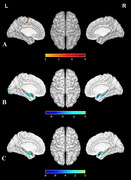

## 1 Introduction

Eczema is a common, chronic, pruritic, and inflammatory skin disease that can cause disruption in the epidermal barrier (David et al. 2017). In addition to cutaneous symptoms, patients with eczema often complain of neurological and psychiatric dysfunctions (J. Kim et al. 2019), such as headaches, insomnia, depression, or anxiety (Buske‐Kirschbaum et al. 2019; Cabanillas et al. 2017). Severe pruritus caused by eczema, in particular can cause an uncomfortable sensation similar to pain (Anzelc and Burkhart 2020; Ji et al. 2019). Pruritus is defined as an uncomfortable sensation that induces the urge to scratch. It is not only the cardinal symptom of eczema but also a common symptom associated with various disorders, such as renal failure (Scherer et al. 2017; Mettang and Kremer 2015) and liver disease (Yoshikawa et al. 2021; Carey et al. 2015), however, the central mechanism that activates neurons associated with pruritus remains largely unknown. The effect of pruritus on the central nervous system (CNS) has long been the focus of research with previous studies simulating the sensation of pruritus via mechanical (e.g. brush) (Hill et al. 2022; Stumpf et al. 2013) or chemical (e.g. histamine, *Lathyrus sativus*, and capsaicin) methods on animal models or humans (Khasabov et al. 2020; Pavlenko et al. 2020; Pan et al. 2019; Zhang et al. 2022; Vierow et al. 2015), to observe the responses within the CNS, including changes in various neurotransmitters or metabolites (Moore et al. 2018). Nevertheless, these methods often fail to provide insights into the persistent effects of pruritus on the brain, especially the functional plasticity in neural circuits related to pruritus (Chen et al. 2021; Chen and Sun 2020).

Resting‐state functional magnetic resonance imaging (rs‐fMRI) is based on the blood oxygen level‐dependent (BOLD) signal (S. Kim and Ogawa 2012) and is a non‐invasive approach for analyzing the characteristics of CNS, which can be used to observe the changes in neural activity in real‐time (Ugurbil 2012). Through indicators, including fraction amplitude of low‐frequency fluctuation (fALFF) (Zou et al. 2008) and functional connectivity (FC) (Biswal et al. 1995), neuronal activities and networks caused by pruritus could be observed. Previous fMRI studies have shown that a pruritic stimuli elicited a significant activation pattern in specific regions within the brain (Sutaria et al. 2022), including the primary somatosensory cortical (S1), the secondary somatosensory cortex (S2), and supplementary motor areas (SMA) (Dong and Dong 2018). Areas involved in emotional processing and evaluation (e.g. cingulate and insula) (Zhang et al. 2022; Li et al. 2021) were also activated during itching and scratching (Liu et al. 2020). Similar to yawning, pruritus is a kind of socially contagious behavior and is prevalent in humans and highly social animals (Mu et al. 2017). Particularly, several areas within the brain associated with pruritus, including the S1, S2, SMA, and thalamus, were activated when the subjects were shown videos where a character demonstrated scratching behaviors (Schut et al. 2015). Interestingly, a similar finding has also been demonstrated in mice (Y. Yu et al. 2017). c‐Fos is commonly used as a biomarker of neuronal activity. The mice that displayed the contagious scratching behavior exhibited a significant increase in c‐Fos expression in the suprachiasmatic nucleus (SCN), the nucleus accumbens, the caudate, the putamen, and in the amygdala (Ehling et al. 2018). Moreover, the above brain regions interacted with each other and established neural circuits, which indicated that the neuroplasticity in patients who suffered from pruritus was significantly altered (Setsu et al. 2021).

These varied findings for both humans and animals may be affected by methodologies and sample selections. Moreover, regional and circuit changes within the brain may indicate an association with different clinical symptoms of patients with pruritus (Sanders and Akiyama 2018). While the relationship between regional and network functional changes remains unclear, specific regions or networks may potentially be used as biomarkers and therapeutic targets for pruritus. Therefore, the purpose of this study was to conduct fMRI studies on patients who suffer from pruritus and explore the relationship between the changes in cerebral activities and clinical manifestations. Based on the above research background, we first hypothesized that brain function changes in response to pruritus are different at the regional or network levels; second, the activity changes in a given brain region may be correlated with the clinical data of patients.

## 2 Materials and Methods

### 2.1 Participants

A total of 42 patients with eczema were enrolled in the PE group. Total 42 healthy controls, who matched for age, gender, years of education, and handedness with the PE group, were recruited from the community as the HC group. All subjects completed fMRI scans between July 2022 and October 2022 from Shuguang Hospital affiliated to Shanghai University of Traditional Chinese Medicine. All experimental protocols were approved by the Institutional Review Board of Shuguang Hospital affiliated to Shanghai University of Traditional Chinese Medicine (Approve Number: 2022‐1168‐105‐01). Their personal data were kept confidential, and they were allowed to withdraw at any time during the prospective study. “Informed consent was obtained from all subjects and/or their legal guardian(s).”

### 2.2 Inclusion/Exclusion Criteria

#### 2.2.1 Diagnostic Criteria for Eczema

(1) Multiform skin lesions with intensely pruritic, erythema and edema as the essential features. (2) Erythematous papules and vesicles with exudation, crusting, erosion, blisters, and oozing. (3) Center of the lesion is more severe, with scattered papules and vesicles in the periphery, the boundary is unclear.

#### 2.2.2 Inclusion Criteria for PE Group

(1) Clinically consistent with the manifestations of eczema, with the first onset and the course of disease within 6 weeks. (2) Between the ages of 18 and 55. (3) Right‐handed; high school education or above. (4) Agree to participate in the study and sign the informed consent.

#### 2.2.3 Inclusion Criteria for HC Group

(1) Previous good health, no history of eczema or pruritus within half a year. (2) Between the ages of 18 and 55. (3) Right‐handed; high school education or above. (4) Agree to participate in the study and sign the informed consent.

#### 2.2.4 Exclusion Criteria for PE and HC Groups

(1) Have had other types of eczema or primary and secondary diseases that can cause pruritus, such as asthma, seborrheic dermatitis, urticaria, cholestasis, uremia, diabetes, and multiple sclerosis. (2) Complicated with cardiovascular and cerebrovascular diseases, liver and kidney insufficiency, hematopoietic system, and mental diseases. (3) Pregnant or lactating women. (4) Oral administration of antihistamines, steroids, and other immunosuppressive agents within 1 week before enrollment. (5) Patients with conditions that are not suitable for MRI examination, such as hearing aid implantation, cardiac pacemaker, claustrophobia, and so on.

### 2.3 Clinical Evaluations Scale

Demographic data were collected for all participants. The Visual Analogue Score (VAS), 12‐Item Pruritus Severity Scale (12‐PSS), Pittsburgh Sleep Quality Index (PSQI), and Self‐Rating Anxiety Scale (SAS) were recorded for the PE group after rs‐fMRI scanning. All instruments were conducted by a trained clinician under strict guidelines and protocols.

### 2.4 Rs‐fMRI Scanning

Rs‐MRI images were acquired by using a 3.0‐Tesla scanner (Siemens MAGNETOM Skyra platform) with a 16‐channel flexible head coil. A sponge‐built head holder was used to prevent head movements. The parameters were set as follows: 3D‐T1WI sequence structural imaging was performed with a magnetization‐prepared rapid gradient echo (MP‐RAGE). TR = 7.2 ms, TE = 3.1 ms, thickness = 1 mm, flip angle = 10°, FOV = 256 × 256 mm, and 192 slices. BOLD‐fMRI images were acquired with a single‐shot gradient recalled echo planar imaging (EPI) sequence. TR = 2000 ms, TE = 30 ms, thickness = 3.5 mm, flip angle = 90°, FOV = 224 × 224 mm, 33 slices, and matrix = 64 × 64. BOLD‐fMRI scan lasted for 8 min, with a total of 240 time points. Participants were instructed to relax with their eyes closed during the scanning process.

### 2.5 Data Processing of Rs‐fMRI

Image preprocessing was performed by SPM 12 (https://fil.ion.ucl.ac.uk/spm) based on Matlab2019b (mathworks.com) platform, and the data involving the following main steps: (1) The first 10 volumes of each scan were removed to avoid instability due to T1‐related relaxation effect. (2) Slice timing corrections: the time difference between data at each point in time and obtain the head motion parameters of the subject in the scanning time series. (3) Realigning: the data at all time points were spatially aligned with the data collected at the first time point to obtain the head motion parameters of the subject in the scanning time series. (4) Coregister and normalization: all the collected data were resampled according to the Montreal Neurological Institute (MNI) standard template space with a 3 × 3 × 3 mm voxel size for spatial normalization. (5) Voxel‐wise detrending: mean signals from white matter and CSF were regressed out, leaving the gray matter signal for denoising. (6) Filtering: the band‐pass filtering range was set at 0.01–0.08 Hz to physiological and high‐frequency noise. (7) Smooth: a Gaussian kernel of 6 mm full width at half‐maximum (FWHM) was used to smooth the images.

The whole‐brain fALFF and FC values were conducted by data processing and analysis of brain imaging (DPABI, http://rfmri.org/dpabi). The fALFF was the ratio of the power spectrum in the low‐frequency band (0.01–0.08 Hz) to the entire frequency range. The fALFF value of each voxel was divided by the global mean fALFF value for each participant to reduce the global effects. For FC analysis, the regions of interest (ROI) based on the comparison results of fALFF values between PE group and HC group were selected. The time series of all voxels in the ROI of each participant were averaged, and the Pearson correlation coefficient between the ROI time series of each participant and the time series of all voxels in the whole brain was calculated to obtain the z‐score graph of FC.

### 2.6 Statistical Analysis

Statistical analysis was performed using SPSS 25.0 version (http://www.spss.com). The results were expressed as mean ± standard deviation. The demographic data and clinical variables (except for gender) were compared using independent‐sample *t*‐test, and gender was compared using chi‐square test. *p* < 0.05 were considered to indicate statistical significance. The fALFF statistical across groups were performed using a voxel‐based, independent‐sample *t*‐test with FDR corrections (*p* < 0.001, cluster size > 50). Brain regions that exhibited differences between the two groups were further elected as ROIs for FC analysis. Mean FC values were extracted within each of these ROIs for further analysis. Furthermore, Pearson correlation coefficients were computed between the extracted fALFF and FC values within these ROIs and the clinical assessments of patients, the significance level was set at *p* < 0.05 (two‐tailed).

## 3 Results

### 3.1 Demographic and Clinical Characteristics of Participants

A total of 84 participants (PE group = 42; HC group = 42) were recruited in the final data analysis. All participants completed the fMRI scanning, and no significant difference in age, sex ratio, and years of education was identified between both groups (*p* > 0.05). Demographic data for all the participants are shown in Table 1.

**TABLE 1:** Demographic and clinical characteristics of participants.

	PE group (*n* = 42)	HC group (*n* = 42)	*p* value
Age (year)	36.81 ± 12.12	34.82 ± 9.96	0.41^a^
Gender (male/female)	14/28	16/26	0.77^b^
Education (year)	14.67 ± 2.26	14.27 ± 2.86	0.48^a^
VAS	6.43 ± 1.21	NA	—
12‐PSS	13.33 ± 3.45	NA	—
PSQI	7.57 ± 4.26	NA	—
SAS	50.02 ± 15.93	NA	—

Abbreviations: HC group: healthy controls; NA: not applicable; PE group: patients with eczema; PSQI: Pittsburgh Sleep Quality Index; SAS: Self‐Rating Anxiety Scale; VAS: Visual Analogue Score; 12‐PSS: 12‐Item Itch Severity Scale.

^a^ Independent‐sample test.

^b^ chi‐square test.

### 3.2 Comparison of fALFF and FC Results Between the Two Groups

**FIGURE 1:**
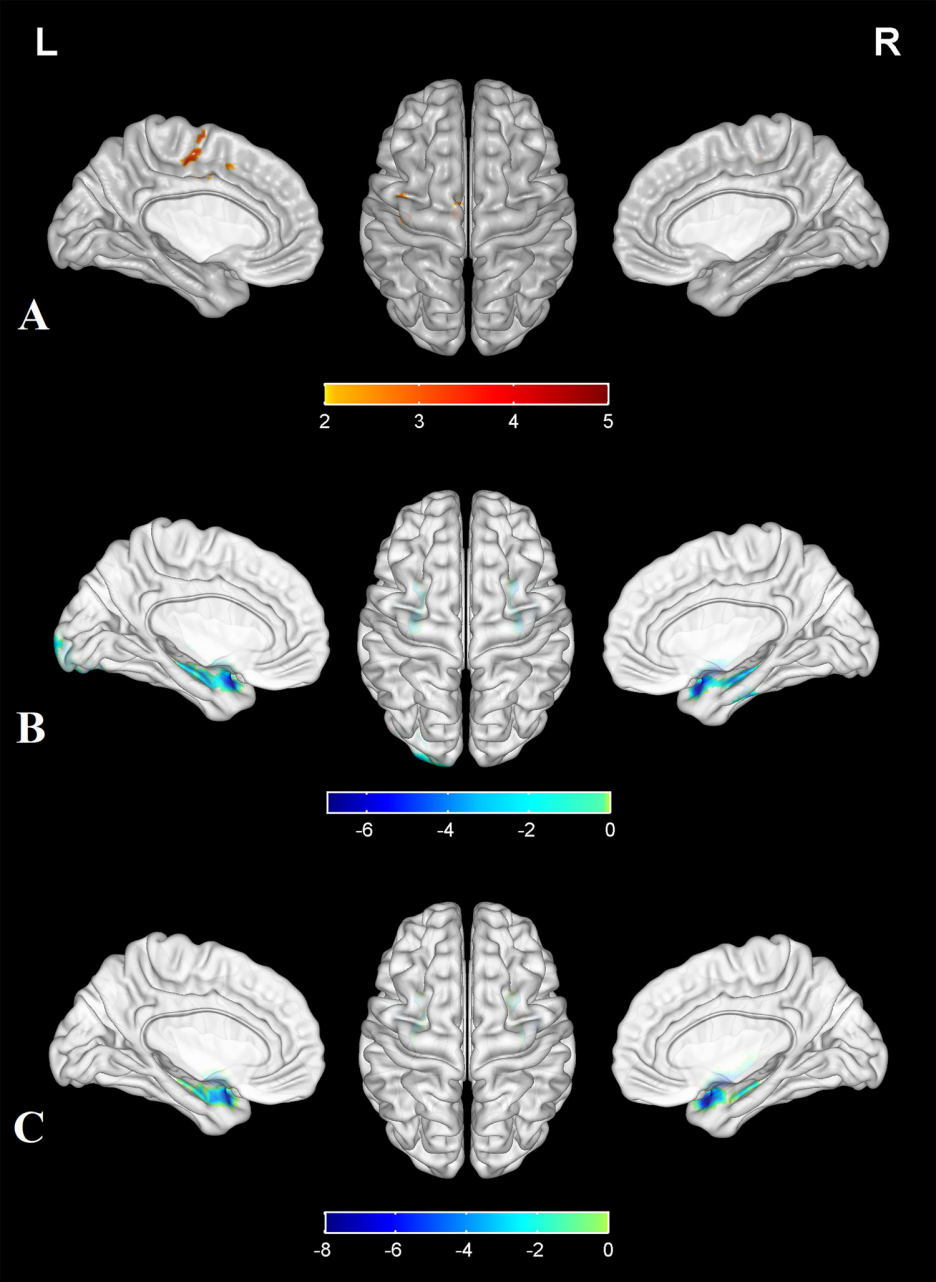
(A) Compared with the HC group, the fALFF values of the left precentral gyrus, left postcentral gyrus, left supplementary motor area (SMA), and left midcingulate cortex (MCC) in the PE group were increased, while no significant decrease in brain was found. (B) Compared with the HC group, the PE group showed decreased FC values between the left precentral gyrus and the following regions: the bilateral inferior temporal gyrus, bilateral hippocampus, and left inferior occipital gyrus. (C) The FC values between left SMA and bilateral inferior temporal gyrus in the PE group were decreased. The colored brain regions with red–yellow indicate a significantly increased fALFF value in the PE group compared with the HC group. The colored brain regions with blue–green indicate a significantly decreased FC value in the PE group compared with the HC group.

Compared with the HC group, the fALFF values of the left precentral gyrus, left postcentral gyrus, left SMA, and left midcingulate cortex (MCC) in the PE group were increased, while no significant decrease in the whole brain was found (Table 2, Figure 1A).

**TABLE 2:** Differences in fALFF and FC values between PE and HC groups.

Brain area	MNI coordinates			Voxels	*t* value^*^	
	X	Y	Z			
fALFF						
Left precentral gyrus	−42	−6	51	95	3.93	
Left postcentral gyrus	−42	−23	49	68	3.66	
Left supplementary motor area	−9	−9	66	96	4.14	
Left midcingulate cortex	−4	35	14	55	3.63	
FC (seed based in left precentral gyrus)						
Left superior temporal gyrus	−33	6	−24	58	−5.92	
Right superior temporal gyrus	36	9	−24	61	−6.10	
Left hippocampus	−25	−21	−10	72	−5.66	
Right hippocampus	29	−20	−10	85	−5.19	
Left inferior occipital gyrus	−39	−90	−15	74	−5.03	
FC (seed based in left SMA)						
Left superior temporal gyrus	−39	3	−24	57		−6.76
Right superior temporal gyrus	36	6	−24	70		−7.50

* voxel‐based, independent‐sample *t*‐test with FDR corrections (*p* < 0.001, cluster size > 50).

The selected FC seeds were based on the fALFF results (left precentral gyrus, left postcentral gyrus, left SMA, and left MCC). Compared with the HC group, the PE group exhibited decreased FC values between the left precentral gyrus and the following regions: the bilateral inferior temporal gyrus, bilateral hippocampus, and left inferior occipital gyrus (Table 2, Figure 1B). The FC values between left SMA and bilateral inferior temporal gyrus in the PE group were decreased (Table 2, Figure 1C). There was no significant difference in FC values between the left postcentral gyrus, the left MCC, and the whole brain.

### 3.3 Correlation Analysis Between fMRI Results and Clinical Scale Data

**FIGURE 2:**
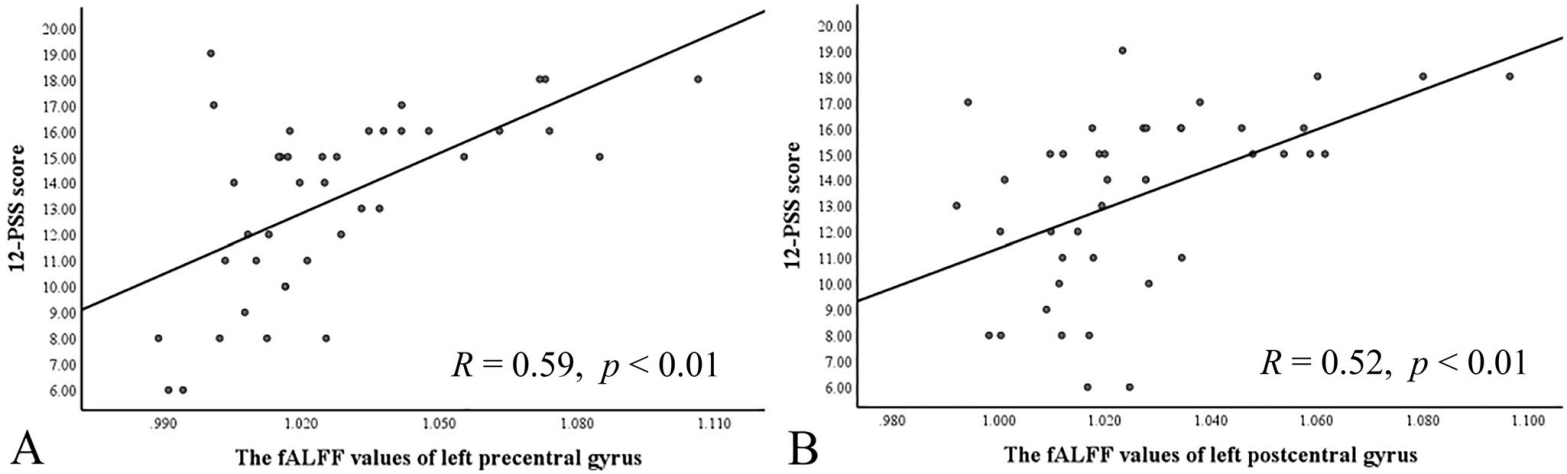
(A) The fALFF values of the left precentral gyrus in the PE group were positively correlated with 12‐PSS score (*R* = 0.59, *p* < 0.01); (B) The fALFF values of left postcentral gyrus in the PE group were positively correlated with 12‐PSS score (*R* = 0.52, *p* < 0.01).

The fALFF values of the left precentral gyrus (*R* = 0.59, *p* < 0.01) and the left postcentral gyrus (*R* = 0.52, *p* < 0.01) in the PE group were positively correlated with 12‐PSS score (Figure 2).

## 4 Discussion

### 4.1 The fMRI Study and Cerebral Regional Activity Characteristic in Patients of Itch

For this study, a special group of patients was selected in the clinic. The selected patients were Koreans without a prior history of eczema in South Korea, however developed eczema accompanied by different degrees of pruritus after living in China for a period of time. The pruritus in these patients was not simulated for experimental purposes which made them the ideal subjects as their pruritus was long standing. Similar to other senses such as pain, temperature, and touch, pruritus was considered to be an independent sensory form that evokes a desire to scratch which is initiated in a particular dermatome and transmitted to the CNS (Sutaria et al. 2022; Darsow et al. 2012). While given that there are particular cerebral regions responsible for processing pruritus, the actual circuits and interconnecting networks related to it in the human brain remains largely unexplored.

Pruritus consists of sensory, emotional, and motivational components (Silverberg 2019). In this fMRI study, we observed that the fALFF values of left MCC, left precentral gyrus, left postcentral gyrus, and left SMA in the PE group were increased. The postcentral gyrus is a critical cortical region for the sensory component of pruritus processing (Spiegel et al. 2017). According to previous brain imaging study, subjects showed significant activation of the postcentral gyrus in response to pruritic stimuli induced by a histamine or saline injection (Woo et al. 2022). In addition, participants who were shown video clips of scratching also demonstrated activation in similar cerebral regions (Najafi et al. 2020). The precentral gyrus, SMA, and cingulate cortex depicts a motor intention from the urge to scratch, particularly, the precentral gyrus and MCC play a key role in driving scratching behavior and are activated by both pruritus and pain (Kakigi and Mochizuki 2011). Numerous studies have shown that cingulate cortex can be activated in both acute pain and chronic pain, and it is associated with emotional expressions such as anxiety and depression during the pain process (Lu et al. 2018). Pruritus and pain share many common targets and neurophysiological bases in terms of regulatory mediators and pathogenesis, such as protease‐activated receptor 2 (PAR2) (Mollanazar et al. 2016), transient receptor potential (TRP) (Moore et al. 2018), and nerve growth factor (NGF) (Indo 2010) activation of peripheral nerve endings, which are the pathological mechanisms of pruritus and pain (Forster and Handwerker 2014). Therefore, we speculate that the abnormal activation of MCC may be related to the experience of itch‐induced anxiety. Considering that the subjects were all right‐handed and all of the activated brain areas were in the left hemisphere, we speculate that the changes may be related to scratching caused by pruritus, and suggested that itch could lead to increased spontaneous activity of the dominant cerebral hemisphere.

### 4.2 The Influence of Cerebral Regional Functional Changes on the Network in Patients With Pruritus

Previous brain imaging studies in healthy subjects have revealed that many brain regions, such as SMA, prefrontal cortex (PFC), insula, thalamus, and cingulate cortex are involved in the perception and processing of pruritus (Vierow et al. 2015; Chen and Sun 2020; Mu et al. 2017). Prior to this study, it was unclear whether there were regional changes caused by the over stimulation from pruritus and if this lead to alterations in neural networks. In our study, FC analysis based on the fALFF results showed widespread decreased FC, mainly involving the superior temporal gyrus, hippocampus, and the inferior occipital gyrus, which provided evidence for the impaired cortical functional in patients with pruritus and implied that regional function alterations exerted considerable effects on network function. The superior temporal gyrus exhibits diverse functions, encompassing higher‐order perception and cognition, as well as visual‐auditory integration. It is closely associated with emotion recognition, facial expression processing, memory, and attention regulation (M. Yu et al. 2022; Chang et al. 2015; Lin et al. 2020).

The hippocampus is involved in spatial orientation, in addition to its well‐established role in memory and learning (Burgess et al. 2002). Furthermore, the hippocampus exhibits close associations with the brain's reward circuitry, and the act of scratching, which serves to alleviate or suppress pruritus, engages the activation or suppression of the reward system. Previous studies have demonstrated the activation of motor‐related regions and reward circuitry (Mochizuki et al. 2015), including the SMA, prefrontal cortex (PFC), and cingulate cortex, during both pruritus and itch‐scratching tasks. Conversely, pruritus‐induced scratching has been found to inhibit the activity of the temporal lobe and hippocampus (H. Kim et al. 2016). The inferior occipital gyrus is a vital component of the default mode network. It is implicated in the regulation of cognitive control, emotional experiences, encoding and integration of memories, as well as self‐awareness and bodily perception (Sellal 2022). Itch sensation is associated with negative emotion (e.g. stress, anxiety, and depress). The reported inferior occipital gyrus may be associated with visual memory and attention deficits in depression (Couvy‐Duchesne et al. 2018), coupled to increased attention and response in expression toward sadness (Palejwala et al. 2021). The decreased FC values in the above brain areas may ultimately point to the remodeling or inhibition of neural function in the itch‐related brain area and the reward system. In order to explore the potential relationship between the clinical manifestations of pruritus and regional/network function alterations, fALFF and FC values of significant brain areas were extracted and correlated with pruritus‐related scales. We found positive correlation between fALFF of the left precentral gyrus, left postcentral gyrus, and 12‐PSS, which suggested that increased fALFF was correlated with the manifestation of pruritus. The direct relationship between regional cerebral function and the pruritus index strengthens the hypothesis of neuroplasticity. Therefore, our study suggests that higher cognitive abilities such as learning, memory, spatial orientation, and facial recognition may be affected in patients with pruritus. Although we found various regional and network abnormalities with increases or decreases in brain functional changes by fMRI in eczema patients with pruritus, there are still some limitations that should be considered in this study. First, this cross‐sectional study hardly reflect the dynamic functional abnormalities during the progression of pruritus. Second, there were more female participants than males both in PE and HC group which may bias our results with more female features. Finally, we did not take into account the confounding effects on brain activity from medications. A larger sample size with more homogenous gender ratios and a longitudinal study, including a comparison before and after treatment, as well as a variety of pruritus‐related serological indicators are needed in future studies to display the progression of brain alterations in patients with itch (Wang et al. 2021).

In conclusion, this study revealed that there was a decrease in spontaneous activity in particular regions within the brain that were affected by itch and there were cerebral functional network connection changes based on the findings from fALFF and FC. There was an abnormal amount of increased regional activity mostly involved in sensorimotor and MCC. Network alterations with widespread FC decrease involved in temporal lobes, occipital lobes, and hippocampus. Our results showed that pruritus affects the spontaneous activity of the sensorimotor cortex in patients with eczema, resulting in extensive imbalances in the FC of brain regions such as the temporal, occipital, and hippocampus. These findings enhanced our understanding of the neural circuit mechanisms underlying pruritus.

## Author Contributions

**Tae‐eun Kim**: investigation, writing–original draft, formal analysis. **Jin Li**: writing–original draft, investigation, validation. **Larissa Tao**: writing–review and editing. **Ji‐ming Tao**: funding acquisition. **Xiang‐yu Wei**: writing–review and editing, data curation.

## Ethics Statement

The study compliances with the ethical standards of the responsible committee on human experimentation (Shuguang Hospital affiliated to Shanghai University of traditional Chinese Medicine; Approve Number: 2022‐1168‐105‐01) and with the Helsinki Declaration of 1975, as revised in 2005. “Informed consent was obtained from all subjects and/or their legal guardian(s).”

## Conflicts of Interest

The authors declare no conflicts of interest.

### Peer Review

The peer review history for this article is available at https://publons.com/publon/10.1002/brb3.70415.

## Data Availability

The data which support the conclusions of our study is included within the article.

## References

[brb370415-bib-0001] Anzelc, M. , and C. G. Burkhart . 2020. “Pain and Pruritus: A Study of Their Similarities and Differences.” International Journal of Dermatology 59: 159–164. 31605395 10.1111/ijd.14678

[brb370415-bib-0002] Biswal, B. , F. Z. Yetkin , V. M. Haughton , and J. S. Hyde . 1995. “Functional Connectivity in the Motor Cortex of Resting Human Brain Using Echo‐Planar MRI.” Magnetic Resonance in Medicine 34: 537–541. 8524021 10.1002/mrm.1910340409

[brb370415-bib-0003] Burgess, N. , E. A. Maguire , and J. O'Keefe . 2002. “The Human Hippocampus and Spatial and Episodic Memory.” Neuron 35: 625–641. 12194864 10.1016/s0896-6273(02)00830-9

[brb370415-bib-0004] Buske‐Kirschbaum, A. , K. Trikojat , F. Tesch , et al. 2019. “Altered Hypothalamus‐Pituitary‐Adrenal Axis Function: A Relevant Factor in the Comorbidity of Atopic Eczema and Attention Deficit/Hyperactivity Disorder?” Psychoneuroendocrinology 105: 178–186. 30583940 10.1016/j.psyneuen.2018.12.005

[brb370415-bib-0005] Cabanillas, B. , A. C. Brehler , and N. Novak . 2017. “Atopic Dermatitis Phenotypes and the Need for Personalized Medicine.” Current Opinion in Allergy and Clinical Immunology 17: 309–315. 28582322 10.1097/ACI.0000000000000376 PMC5515628

[brb370415-bib-0006] Carey, E. J. , A. H. Ali , and K. D. Lindor . 2015. “Primary Biliary Cirrhosis.” Lancet 386: 1565–1575. 26364546 10.1016/S0140-6736(15)00154-3

[brb370415-bib-0007] Chang, E. F. , K. P. Raygor , and M. S. Berger . 2015. “Contemporary Model of Language Organization: An Overview for Neurosurgeons.” Journal of Neurosurgery 122: 250–261. 25423277 10.3171/2014.10.JNS132647

[brb370415-bib-0008] Chen, X. J. , Y. H. Liu , N. L. Xu , and Y. G. Sun . 2021. “Multiplexed Representation of Itch and Mechanical and Thermal Sensation in the Primary Somatosensory Cortex.” Journal of Neuroscience 41: 10330–10340. 34716234 10.1523/JNEUROSCI.1445-21.2021 PMC8672683

[brb370415-bib-0009] Chen, X. J. , and Y. G. Sun . 2020. “Central Circuit Mechanisms of Itch.” Nature Communications 11: 3052. 10.1038/s41467-020-16859-5 PMC7297978 32546780

[brb370415-bib-0010] Couvy‐Duchesne, B. , L. T. Strike , G. I. de Zubicaray , et al. 2018. “Lingual Gyrus Surface Area Is Associated With Anxiety‐Depression Severity in Young Adults: A Genetic Clustering Approach.” eNeuro 5, no. 1: ENEURO.0153–0117.2017. 10.1523/ENEURO.0153-17.2017 PMC5773884 29354681

[brb370415-bib-0011] Darsow, U. , F. Pfab , M. Valet , T. R. Tolle , and J. Ring . 2012. “Itch and Eczema.” Chemical Immunology and Allergy 96: 81–88. 22433375 10.1159/000331890

[brb370415-bib-0012] David, B. W. , J. A. Tarbox , and M. B. Tarbox . 2017. “Atopic Dermatitis: Pathophysiology.” Advances in Experimental Medicine and Biology 1027: 21–37. 29063428 10.1007/978-3-319-64804-0_3

[brb370415-bib-0013] Dong, X. , and X. Dong . 2018. “Peripheral and Central Mechanisms of Itch.” Neuron 98: 482–494. 29723501 10.1016/j.neuron.2018.03.023 PMC6022762

[brb370415-bib-0014] Ehling, S. , A. Butler , S. Thi , H. T. Ghashghaei , and W. Baumer . 2018. “To Scratch an Itch: Establishing a Mouse Model to Determine Active Brain Areas Involved in Acute Histaminergic Itch.” IBRO Rep 5: 67–73. 30364768 10.1016/j.ibror.2018.10.002 PMC6197726

[brb370415-bib-0015] Forster, C. , and H. O. Handwerker . 2014. Central Nervous Processing of Itch and Pain. CRC Press. 24830010

[brb370415-bib-0016] Hill, R. Z. , M. C. Loud , A. E. Dubin , B. Peet , and A. Patapoutian . 2022. “PIEZO1 Transduces Mechanical Itch in Mice.” Nature 607: 104–110. 35732741 10.1038/s41586-022-04860-5 PMC9259491

[brb370415-bib-0017] Indo, Y. 2010. “Nerve Growth Factor, Pain, Itch and Inflammation: Lessons From Congenital Insensitivity to Pain With Anhidrosis.” Expert Review of Neurotherapeutics 10: 1707–1724. 20977328 10.1586/ern.10.154

[brb370415-bib-0018] Ji, R. R. , C. R. Donnelly , and M. Nedergaard . 2019. “Astrocytes in Chronic Pain and Itch.” Nature Reviews Neuroscience 20: 667–685. 31537912 10.1038/s41583-019-0218-1 PMC6874831

[brb370415-bib-0019] Kakigi, R. , and H. Mochizuki . 2011. “Mechanisms of Intracerebral Pain and Itch Perception in Humans.” Brain Nerve 63: 987–994. 21878701

[brb370415-bib-0020] Khasabov, S. G. , H. Truong , V. M. Rogness , K. D. Alloway , D. A. Simone , and G. J. Giesler . 2020. “Responses of Neurons in the Primary Somatosensory Cortex to Itch‐ and Pain‐Producing Stimuli in Rats.” Journal of Neurophysiology 123: 1944–1954. 32292106 10.1152/jn.00038.2020 PMC7444912

[brb370415-bib-0021] Kim, J. , B. E. Kim , and D. Leung . 2019. “Pathophysiology of Atopic Dermatitis: Clinical Implications.” Allergy and Asthma Proceedings 40: 84–92. 30819278 10.2500/aap.2019.40.4202 PMC6399565

[brb370415-bib-0022] Kim, S. G. , and S. Ogawa . 2012. “Biophysical and Physiological Origins of Blood Oxygenation Level‐Dependent fMRI Signals.” Journal of Cerebral Blood Flow and Metabolism 32: 1188–1206. 22395207 10.1038/jcbfm.2012.23 PMC3390806

[brb370415-bib-0023] Kim, H. J. , J. B. Park , J. H. Lee , and I. H. Kim . 2016. “How Stress Triggers Itch: A Preliminary Study of the Mechanism of Stress‐Induced Pruritus Using fMRI.” International Journal of Dermatology 55: 434–442. 26276021 10.1111/ijd.12864

[brb370415-bib-0024] Li, X. , J. Yao , K. H. Hu , et al. 2021. “Differential Roles of Prelimbic and Anterior Cingulate Cortical Region in the Modulation of Histaminergic and Non‐Histaminergic Itch.” Behavioural Brain Research 411: 113388. 34052263 10.1016/j.bbr.2021.113388

[brb370415-bib-0025] Lin, Y. H. , I. M. Young , A. K. Conner , et al. 2020. “Anatomy and White Matter Connections of the Inferior Temporal Gyrus.” World Neurosurgery 143: e656–e666. 32798785 10.1016/j.wneu.2020.08.058

[brb370415-bib-0026] Liu, X. , X. H. Miao , and T. Liu . 2020. “More Than Scratching the Surface: Recent Progress in Brain Mechanisms Underlying Itch and Scratch.” Neuroscience Bulletin 36: 85–88. 30830669 10.1007/s12264-019-00352-1 PMC6940400

[brb370415-bib-0027] Lu, Y. C. , Y. J. Wang , B. Lu , M. Chen , P. Zheng , and J. G. Liu . 2018. “ACC to Dorsal Medial Striatum Inputs Modulate Histaminergic Itch Sensation.” Journal of Neuroscience 38: 3823–3839. 29540548 10.1523/JNEUROSCI.3466-17.2018 PMC6705910

[brb370415-bib-0028] Mettang, T. , and A. E. Kremer . 2015. “Uremic Pruritus.” Kidney International 87: 685–691. 24402092 10.1038/ki.2013.454

[brb370415-bib-0029] Mochizuki, H. , A. Papoiu , L. A. Nattkemper , et al. 2015. “Scratching Induces Overactivity in Motor‐Related Regions and Reward System in Chronic Itch Patients.” Journal of Investigative Dermatology 135: 2814–2823. 26076316 10.1038/jid.2015.223

[brb370415-bib-0030] Mollanazar, N. K. , P. K. Smith , and G. Yosipovitch . 2016. “Mediators of Chronic Pruritus in Atopic Dermatitis: Getting the Itch Out?” Clinical Reviews in Allergy & Immunology 51: 263–292. 25931325 10.1007/s12016-015-8488-5

[brb370415-bib-0031] Moore, C. , R. Gupta , S. E. Jordt , Y. Chen , and W. B. Liedtke . 2018. “Regulation of Pain and Itch by TRP Channels.” Neuroscience Bulletin 34: 120–142. 29282613 10.1007/s12264-017-0200-8 PMC5799130

[brb370415-bib-0032] Mu, D. , J. Deng , K. F. Liu , et al. 2017. “A Central Neural Circuit for Itch Sensation.” Science 357: 695–699. 28818946 10.1126/science.aaf4918

[brb370415-bib-0033] Najafi, P. , S. D. Ben , J. L. Carre , L. Misery , and O. Dufor . 2020. “Functional and Anatomical Brain Connectivity in Psoriasis Patients and Healthy Controls: A Pilot Brain Imaging Study After Exposure to Mentally Induced Itch.” Journal of the European Academy of Dermatology and Venereology 34: 2557–2565. 32267024 10.1111/jdv.16441

[brb370415-bib-0034] Palejwala, A. H. , N. B. Dadario , and I. M. Young , et al. 2021. “Anatomy and White Matter Connections of the Lingual Gyrus and Cuneus.” World Neurosurgery 151: e426–e437. 33894399 10.1016/j.wneu.2021.04.050

[brb370415-bib-0035] Pan, H. , M. Fatima , A. Li , et al. 2019. “Identification of a Spinal Circuit for Mechanical and Persistent Spontaneous Itch.” Neuron 103: 1135–1149. 31324538 10.1016/j.neuron.2019.06.016 PMC6763390

[brb370415-bib-0036] Pavlenko, D. , H. Funahashi , K. Sakai , et al. 2020. “IL‐23 Modulates Histamine‐Evoked Itch and Responses of Pruriceptors in Mice.” Experimental Dermatology 29: 1209–1215. 33010057 10.1111/exd.14206

[brb370415-bib-0037] Sanders, K. M. , and T. Akiyama . 2018. “The Vicious Cycle of Itch and Anxiety.” Neuroscience and Biobehavioral Reviews 87: 17–26. 29374516 10.1016/j.neubiorev.2018.01.009 PMC5845794

[brb370415-bib-0038] Scherer, J. S. , S. A. Combs , and F. Brennan . 2017. “Sleep Disorders, Restless Legs Syndrome, and Uremic Pruritus: Diagnosis and Treatment of Common Symptoms in Dialysis Patients.” American Journal of Kidney Diseases 69: 117–128. 27693261 10.1053/j.ajkd.2016.07.031 PMC5497466

[brb370415-bib-0039] Schut, C. , S. Grossman , U. Gieler , J. Kupfer , and G. Yosipovitch . 2015. “Contagious Itch: What We Know and What We Would Like to Know.” Frontiers in Human Neuroscience 9: 57. 25717300 10.3389/fnhum.2015.00057 PMC4324061

[brb370415-bib-0040] Sellal, F. 2022. “Anatomical and Neurophysiological Basis of Face Recognition.” Revue Neurologique 178: 649–653. 34863530 10.1016/j.neurol.2021.11.002

[brb370415-bib-0041] Setsu, T. , Y. Hamada , D. Oikawa , et al. 2021. “Direct Evidence That the Brain Reward System Is Involved in the Control of Scratching Behaviors Induced by Acute and Chronic Itch.” Biochemical and Biophysical Research Communications 534: 624–631. 33220930 10.1016/j.bbrc.2020.11.030

[brb370415-bib-0042] Silverberg, J. I. 2019. “Comorbidities and the Impact of Atopic Dermatitis.” Annals of Allergy, Asthma & Immunology 123: 144–151. 31034875 10.1016/j.anai.2019.04.020

[brb370415-bib-0043] Spiegel, D. R. , A. Pattison , A. Lyons , et al. 2017. “The Role and Treatment Implications of Peripheral and Central Processing of Pain, Pruritus, and Nausea in Heightened Somatic Awareness: A Review.” Innovations in Clinical Neuroscience 14: 11–20. PMC5605199 28979822

[brb370415-bib-0044] Stumpf, A. , M. Burgmer , G. Schneider , et al. 2013. “Sex Differences in Itch Perception and Modulation by Distraction–An FMRI Pilot Study in Healthy Volunteers.” PLoS ONE 8: e79123. 24260163 10.1371/journal.pone.0079123 PMC3832610

[brb370415-bib-0045] Sutaria, N. , W. Adawi , R. Goldberg , Y. S. Roh , J. Choi , and S. G. Kwatra . 2022. “Itch: Pathogenesis and Treatment.” Journal of the American Academy of Dermatology 86: 17–34. 34648873 10.1016/j.jaad.2021.07.078

[brb370415-bib-0046] Ugurbil, K. 2012. “Development of Functional Imaging in the Human Brain (fMRI); the University of Minnesota Experience.” Neuroimage 62: 613–619. 22342875 10.1016/j.neuroimage.2012.01.135 PMC3530260

[brb370415-bib-0047] Vierow, V. , C. Forster , R. Vogelgsang , A. Dorfler , and H. O. Handwerker . 2015. “Cerebral Networks Linked to Itch‐Related Sensations Induced by Histamine and Capsaicin.” Acta Dermato‐Venereologica 95: 645–652. 25387448 10.2340/00015555-2006

[brb370415-bib-0048] Wang, Z. , C. Jiang , H. Yao , et al. 2021. “Central Opioid Receptors Mediate Morphine‐Induced Itch and Chronic Itch via Disinhibition.” Brain 144: 665–681. 33367648 10.1093/brain/awaa430

[brb370415-bib-0049] Woo, S. , Y. R. Kim , M. S. Bak , G. Chung , S. J. Kim , and S. K. Kim . 2022. “Multiplexed Representation of Itch and Pain and Their Interaction in the Primary Somatosensory Cortex.” Experimental Neurobiology 31: 324–331. 36351842 10.5607/en22029 PMC9659493

[brb370415-bib-0050] Yoshikawa, S. , T. Asano , M. Morino , et al. 2021. “Pruritus Is Common in Patients With Chronic Liver Disease and Is Improved by Nalfurafine Hydrochloride.” Scientific Reports 11: 3015. 33542298 10.1038/s41598-021-82566-w PMC7862656

[brb370415-bib-0051] Yu, Y. Q. , D. M. Barry , Y. Hao , X. T. Liu , and Z. F. Chen . 2017. “Molecular and Neural Basis of Contagious Itch Behavior in Mice.” Science 355: 1072–1076. 28280205 10.1126/science.aak9748 PMC5502115

[brb370415-bib-0052] Yu, M. , Y. Song , and J. Liu . 2022. “The Posterior Middle Temporal Gyrus Serves as a Hub in Syntactic Comprehension: A Model on the Syntactic Neural Network.” Brain and Language 232: 105162. 35908340 10.1016/j.bandl.2022.105162

[brb370415-bib-0053] Zhang, T. T. , S. S. Guo , H. Y. Wang , et al. 2022. “An Anterior Cingulate Cortex‐to‐Midbrain Projection Controls Chronic Itch in Mice.” Neuroscience Bulletin 39: 793–807. 36528690 10.1007/s12264-022-00996-6 PMC10169993

[brb370415-bib-0054] Zou, Q. H. , C. Z. Zhu , Y. Yang , et al. 2008. “An Improved Approach to Detection of Amplitude of Low‐Frequency Fluctuation (ALFF) for Resting‐State fMRI: Fractional ALFF.” Journal of Neuroscience Methods 172: 137–141. 18501969 10.1016/j.jneumeth.2008.04.012 PMC3902859

